# Patient reported barriers are associated with low physical and mental well-being in patients with co-morbid diabetes and chronic kidney disease

**DOI:** 10.1186/s12955-018-1044-2

**Published:** 2018-11-19

**Authors:** Edward Zimbudzi, Clement Lo, Sanjeeva Ranasinha, Gregory Fulcher, Martin Gallagher, Stephen Jan, Peter G. Kerr, Helena J. Teede, Kevan R. Polkinghorne, Grant Russell, Rowan G. Walker, Sophia Zoungas

**Affiliations:** 1 School of Public Health and Preventive Medicine, Monash University, Melbourne, VIC Australia; 2 Department of Nephrology, Monash Health, Melbourne, VIC Australia; 3 Diabetes and Vascular Medicine Unit, Monash Health, Melbourne, VIC Australia; 4 Department of Diabetes and Endocrinology, Royal North Shore Hospital, Sydney, NSW Australia; 5 Northern Clinical School, University of Sydney, Royal North Shore Hospital, Sydney, NSW Australia; 6 The George Institute for Global Health, University of NSW, Sydney, NSW Australia; 7 Concord Clinical School, University of Sydney, Sydney, NSW Australia; 8 Sydney Medical School, University of Sydney, Sydney, NSW Australia; 9 Department of Medicine, School of Clinical Sciences at Monash Health, Monash University, Melbourne, VIC Australia; 10 School of Primary Health and Allied Health Care, Monash University, Melbourne, VIC Australia; 11 Department of Renal Medicine, Alfred Hospital, Melbourne, VIC Australia

**Keywords:** Chronic kidney disease, Diabetes, Health related quality of life, Mental well-being, Patient reported barriers, Physical well-being

## Abstract

**Background:**

Little is known about how patient reported barriers to health care impact the quality of life (HRQoL) of patients with comorbid disease. We investigated patient reported barriers to health care and low physical and mental well-being among people with diabetes and chronic kidney disease (CKD).

**Methods:**

Adults with diabetes and CKD (estimated Glomerular Filtration Rate < 60 ml/min/1.73m^2^) were recruited and completed a questionnaire on barriers to health care, the 12-Item HRQoL Short Form Survey and clinical assessment. Low physical and mental health status were defined as mean scores < 50. Logistic regression models were used.

**Results:**

Three hundred eight participants (mean age 66.9 ± 11 years) were studied. Patient reported ‘impact of the disease on family and friends’ (OR 2.07; 95% CI 1.14 to 3.78), ‘feeling unwell’ (OR 4.23; 95% CI 1.45 to 12.3) and ‘having other life stressors that make self-care a low priority’ (OR 2.59; 95% CI 1.20 to 5.61), were all associated with higher odds of low physical health status. Patient reported ‘feeling unwell’ (OR 2.92; 95% CI 1.07 to 8.01), ‘low mood’ (OR 2.82; 95% CI 1.64 to 4.87) and ‘unavailability of home help’ (OR 1.91; 95% CI 1.57 to 2.33) were all associated with higher odds of low mental health status. The greater the number of patient reported barriers the higher the odds of low mental health but not physical health status.

**Conclusions:**

Patient reported barriers to health care were associated with lower physical and mental well-being. Interventions addressing these barriers may improve HRQoL among people with comorbid diabetes and CKD.

**Electronic supplementary material:**

The online version of this article (10.1186/s12955-018-1044-2) contains supplementary material, which is available to authorized users.

## Background

Health-related quality of life (HRQOL) is a multi-dimensional concept commonly used to examine the impact of health status on quality of life [1] and is widely regarded as the best assessment of the impact of disease on a patient’s well-being [2]. Among patients with comorbid diabetes and chronic kidney disease (CKD), low HRQoL [3, 4] as well as its association with several demographic [3, 5] and disease factors has been reported [4, 6], but little is known about its association with patient reported barriers to health care. Examining the patient reported barriers associated with HRQoL offers an excellent opportunity for addressing the provision of patient-centred care, which is largely considered the gold standard for health care across the world [7].

Among patients with diabetes, those who have reported barriers such as cost, transportation difficulties, competing demands, low self-efficacy and psychosocial barriers have also reported lower physical and mental well-being [8, 9]. In contrast, among patients with CKD, the impact of patient reported barriers such as communication, physical health, socioeconomic status, psychosocial and access to health services on physical and mental well-being has not been reported [10]. While patient reported barriers to health care for patients with comorbid diabetes and CKD have been characterised [11], their association with optimal physical or mental well-being is largely unknown.

A comprehensive understanding of key modifiable patient reported barriers to health care may thus inform the development of contextually tailored interventions to improve the physical and mental well-being of patients with comorbid diabetes and CKD. The objective of this study was to explore the association between patient reported barriers to health care and the physical and mental health well-being of patients with diabetes and CKD. We hypothesized that patients with comorbid diabetes and CKD who experience barriers to health care will report lower mental and physical well-being. We also hypothesized that mental and physical well-being would vary depending on the number patient-reported barriers.

## Methods

### Study design, setting and participants

This multi-centre cross-sectional study was conducted across four large tertiary hospitals in Australia’s two most populous cities, (Alfred and Monash Health in Melbourne and the Royal North Shore and Concord Hospitals in Sydney). The study also involved collaboration with research institutes, national consumer stakeholder groups (Diabetes Australia and Kidney Health Australia) and primary care groups.

Adult patients (over 18 years) who were fluent in English and had diabetes and CKD (eGFR < 60 ml/min/1.73m^2^) were drawn from ambulatory diabetes or renal clinics of each participating tertiary hospital between January to September 2014. The diagnosis of diabetes was noted on medical records and/or confirmed by laboratory results as per World Health Organisation (WHO) criteria [12, 13]. Patients were considered to have CKD if they had a sustained estimated glomerular filtration rate (eGFR) < 60 mL/min/1.73 m^2^ calculated using the CKD-EPI (Chronic Kidney Disease Epidemiology Collaboration) equation [14] (i.e. two or more eGFR readings) over a 3 month period.

The reporting in this study followed the STROBE (Strengthening The Reporting of Observational Studies in Epidemiology) guidelines [15]. Ethics approval was obtained from Monash University and respective health service ethics committees.

### Demographic and clinical variables

Age, gender, language spoken at home, socio-economic status (SES), stage of kidney disease, duration of kidney disease and duration of diabetes were obtained from the first questionnaire (see Additional file 1) which was prospectively completed by site study staff or the clinician, using standardised procedures from the doctor’s notes and laboratory results from clinic. We estimated socio-economic status using the Australian Bureau of Statistics data [16]. Postcodes were coded according to the Index of Relative Social Disadvantage (IRSD), a composite measure based on selected census variables, which include income, educational attainment and employment status. The IRSD scores for each postcode were then grouped into quintiles for analysis, where the highest quintile comprised 20% of postcodes with the highest IRSD scores (the most advantaged areas).

### Patient reported barriers

Patients completed the second questionnaire, which examined patient reported barriers to health care (see Additional file 2). The barriers were identified from the content analysis of 12 focus groups of 58 participants with co-morbid diabetes and CKD and 8 semi-structured interviews of carers from a previous multi-centre qualitative study performed by the authors [11]. Patient reported barriers were organised into three categories namely personal, clinician and health system-related barriers.

### Health-related quality of life

The Kidney Disease and Quality of Life (KDQoL™-36) questionnaire [17] (see Additional file 3) measured the physical and mental well-being of patients. The KDQoL-36™ is a is a 36-item survey that includes the SF-12 as generic core plus 24 items on quality of life related to kidney disease (the burden of kidney disease, symptoms/problems of kidney disease, and effects of kidney disease scales). Item scores were summed for each scale and transformed on a scale of 0 to 100 with a higher score indicating better HRQoL. This study utilised the SF-12 physical and mental composite measures, which both have a general population mean of 50 and standard deviation of 10. Scores less than 50 were categorised as low health status. The validity and reliability of the KDQoL-36 questionnaire has been reported previously [18–20].

### Statistical analysis

Distributions of demographic and clinical characteristics are presented as descriptive statistics (continuous variables are reported as means and standard deviations or medians with interquartile ranges if distributions are skewed and categorical variables are reported as frequencies and percentages). First, a sub-analysis according to low and high physical and mental well-being was performed for age, gender, stage of kidney disease, diabetes duration and all the patient reported barriers. Continuous data were analysed with t-tests and categorical data were analysed with chi squared test and Fisher’s exact tests, as appropriate. To analyse barriers, Likert scales were collapsed into 2 categories (disagree and agree). Second, univariable and multivariable logistic regression were performed to identify factors associated with lower physical and mental health well-being. Potential factors included demographic and patient reported barriers to health care. The multivariable model included variables identified a priori to be of importance (age and gender) and factors significant on univariable analyses. Predictor variables with *p* < 0.05 in univariable analyses were included in multivariable models to reduce the likelihood of type 2 error. Statistical significance was indicated by a *p* value of < 0.05 in multivariable analyses. All analyses were performed with Stata version 11 (Statacorp, College Station, TX).

## Results

### Patient characteristics

**Fig. 1:**
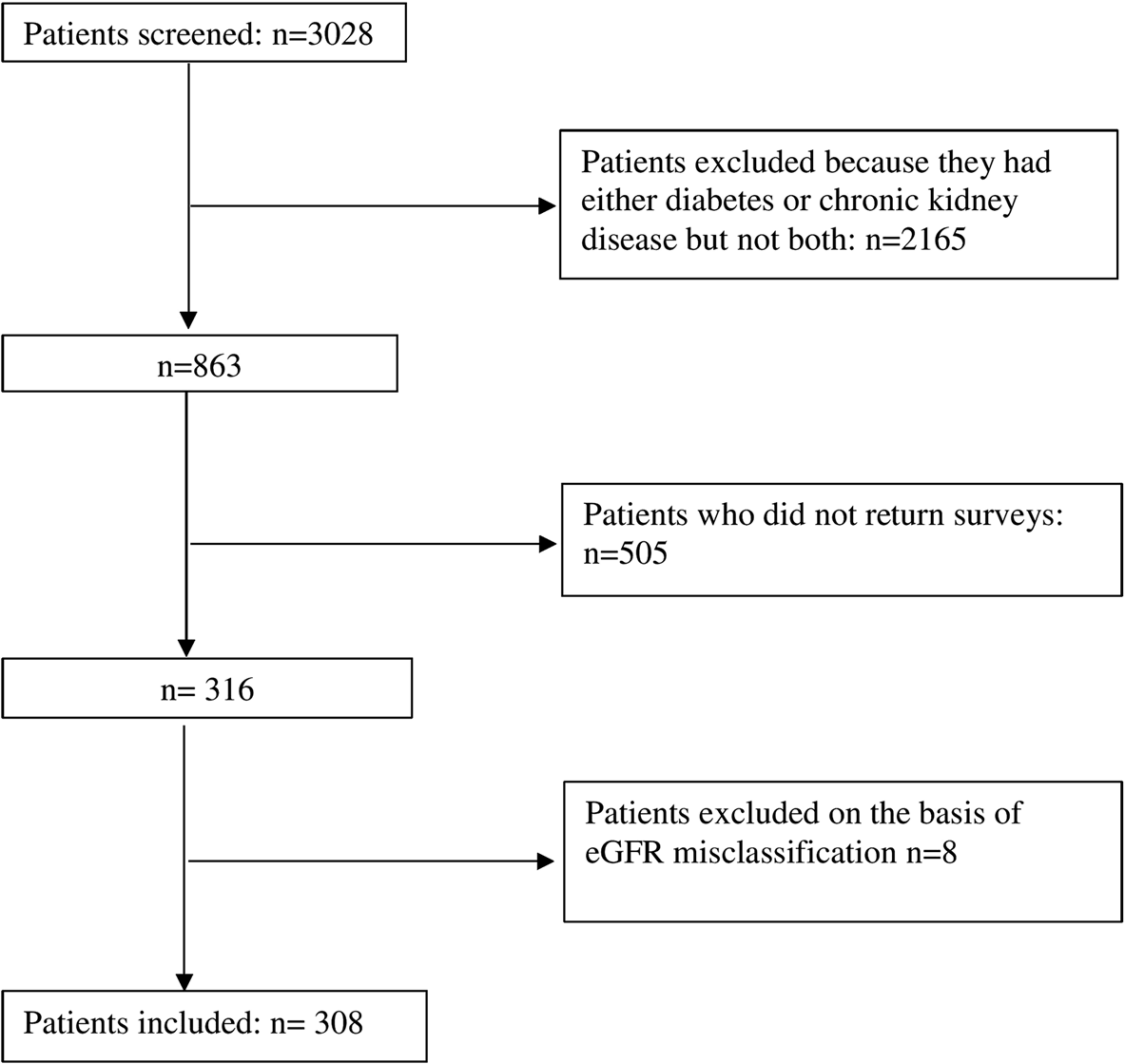
Patient recruitment

Of the 3028 patients identified with diabetes or CKD, 863 met the inclusion criteria and were invited to participate and of these, 308 agreed to participate (Fig. 1). The final inclusion rate based on eligible participants was 36%. Characteristics of respondents and non-respondents are reported in Additional file 4: Table S1. Responders were younger and predominantly male. There were no differences with respect to type of diabetes and stage of kidney disease. The demographic and clinical characteristics of respondents are described in Table 1.

**Table 1:** Baseline characteristics

Variable	Mean ± SD/%	Range
Age (years)	66.9 ± 11.0	32–90
Male (%)	69.5	
Socio-economic status (in quintiles) *		
Lower	20.3	
Upper lower	19.3	
Lower middle	20.0	
Upper middle	21.0	
Upper	19.3	
English speaking (%)	78.0	
Currently smoking (%)	7.8	
Diabetes type (%)		
Type 1	9.1	
Type 2	88.0	
Other	2.9	
Diabetes duration (median, IQR^♦^) years	17 (13)	1–57
CKD stages		
3a	23.4	
b	25.7	
4	24.6	
5 (including dialysis)	26.3	
Health Related Quality of Life		
SF-12 Physical Composite Summary	35.2 ± 11.1	12–64
SF-12 Mental Composite Summary	47.1 ± 10.9	10–68

* Socio-economic status was estimated using the Australian Bureau of Statistics data. Postcodes were coded according to the Index of Relative Social Disadvantage (IRSD), a composite measure based on selected census variables, which include income, educational attainment and employment status. ^♦^IQR-Interquartile range.

The mean age of participants was 66.9 ± 11.0 years, 70% were male and most were English speaking (78%) and evenly distributed across the socio-economic quintiles (lower-20.3%, upper lower-19.3%, lower middle-20.0%, upper middle-21.0% and upper-19.3%). Most had type 2 diabetes (88.0%) with 23.4, 25.7, 24.6 and 26.3% having CKD stage 3a, 3b, 4 and 5 respectively.

### Health related quality of life

The mean ± SD for the physical and mental composite scores were 35.2 ± 11.1 and 47.1 ± 10.9 respectively. The proportions of patients who scored below the general population mean (μ = 50 and SD = 10) for the physical and mental composite scores were 86 and 51% respectively (Table 2).

**Table 2:** Differences between low and high groups on demographic and clinical characteristics

Measure	Physical health status			Mental health status		
	*Low scores (*N* = 158)	High Scores (*N* = 26)	*P*-value	*Low scores (*N* = 94)	High Scores (*N* = 90)	*P*-value
Age (years), mean (SD)	66.9 (11.2)	66.3 (9.5)	0.79	65.5 (11.5)	68.5 (10.2)	0.02
Gender						
Male, n (%)	172 (68.3)	28 (69.6)	0.05	110 (69.2)	90 (71.4)	0.68
Female, n (%)	80 (31.7)	5 (30.4)		49 (30.8)	36 (28.6)	
Socioeconomic status, n (%)						
Lower	51 (20.2)	8 (24.2)	0.97	37 (23.3)	22 (17.4)	0.65
Upper lower	51 (20.2)	7 (21.2)		33 (20.8)	25 (19.8)	
Lower middle	47 (18.7)	6 (18.2)		29 (18.2)	24 (19.0)	
Upper middle	54 (21.4)	7 (21.2)		34 (21.4)	27 (21.4)	
Upper	49 (19.4)	5 (15.2)		26 (16.4)	28 (22.2)	
Language, n (%)						
English speaking	189 (75.9)	31 (94.0)	0.02	117 (74.1)	103 (83.1)	0.08
Non-English speaking	60 (24.1)	2 (6.0)		41 (25.9)	21 (16.9)	
Smoking status, n (%)						
Yes	15 (8.1)	1 (3.8)	0.70	13 (11.3)	3 (3.1)	0.04
No	170 (91.9)	25 (96.2)		102 (88.7)	93 (96.9)	
Diabetes type, n (%)						
Type 1	25 (9.9)	3 (9.1)	1.00	17 (10.7)	11 (8.7)	0.71
Type 2	219 (86.9)	29 (87.9)		136 (85.5)	112 (88.9)	
Other	8 (3.2)	1 (3.0)		6 (3.8)	3 (2.4)	
Diabetes duration (years), median (IQR)	17 (0–57)	19 (1–34)	0.68	16 (0–53)	20 (1–57)	0.13
CKD stages, n (%)						
3a	52 (20.6)	12 (36.4)	0.03	33 (20.8)	31 (24.6)	0.27
3b	62 (24.6)	11 (33.3)		37 (23.3)	36 (28.6)	
4	68 (27.0)	3 (9.1)		39 (24.5)	32 (25.4)	
5	70 (27.8)	7 (21.1)		50 (31.4)	27 (21.4)	

*Scores were defined as low for both physical and mental well-being if they were lower than the general population mean (μ = 50 and SD)

Patients with low physical health status differed by stage of CKD (*p* = 0.03) and language spoken (*p* = 0.02), and patients with low mental health status differed by age (p = 0.02) and smoking status (*p* = 0.04) but not gender, socio-economic status, type of diabetes and duration of diabetes (all *p* > 0.05) (Table 2).

### Patient reported barriers associated with lower physical and mental well-being

**Fig. 2:**
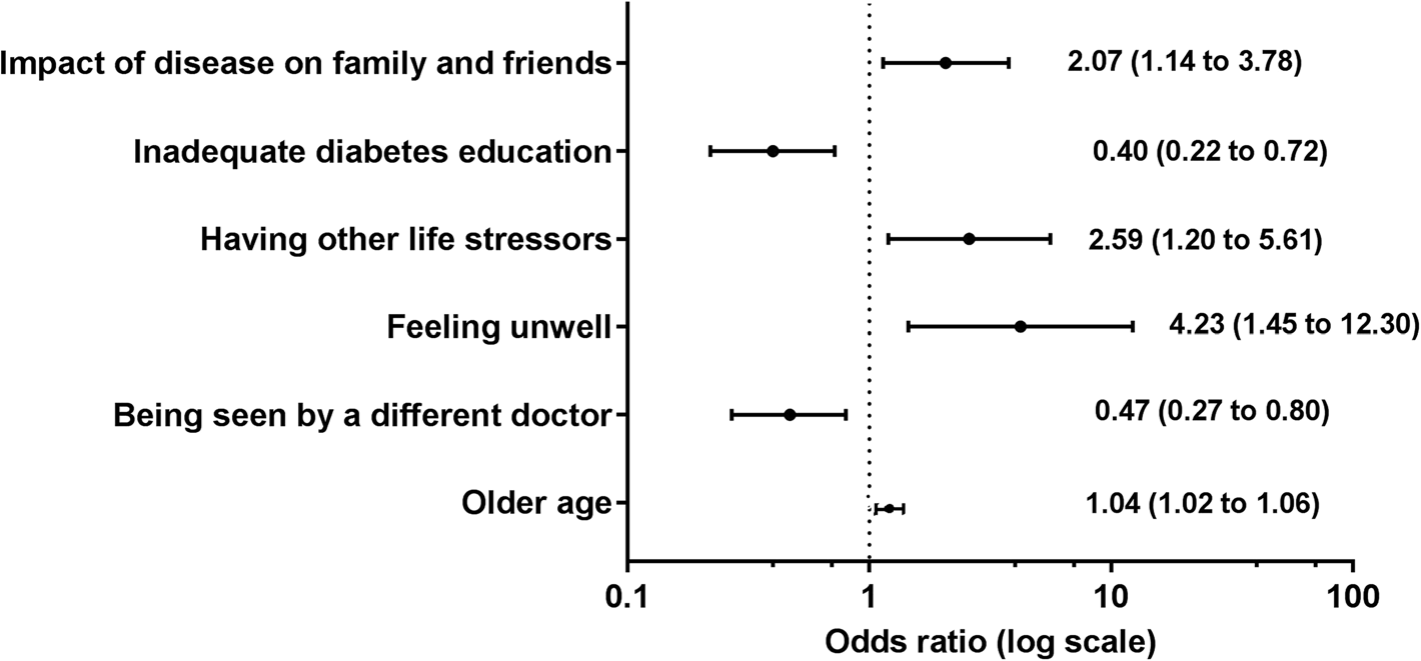
Patient reported barriers associated with low physical health status

Patient reported barriers associated with higher odds of low physical health status included the personal barriers of ‘impact of the disease on family and friends’ (OR 2.07; 95% CI 1.14 to 3.78), ‘feeling unwell’ (OR 4.23; 95% CI 1.45 to 12.3) and ‘having other life stressors that make self-care a low priority’ (OR 2.59; 95% CI 1.20 to 5.61) (Fig. 2 and Additional file 4: Table S2). Patient reported barriers associated with lower odds of low physical health status included the clinician and health system barriers of ‘being seen by a different doctor’ (OR 0.47; 95% CI 0.27 to 0.80) and ‘inadequate diabetes education’ (OR 0.40; 95% CI 0.22 to 0.72) (Fig. 2 and Additional file 4: Table S2).

**Fig. 3:**
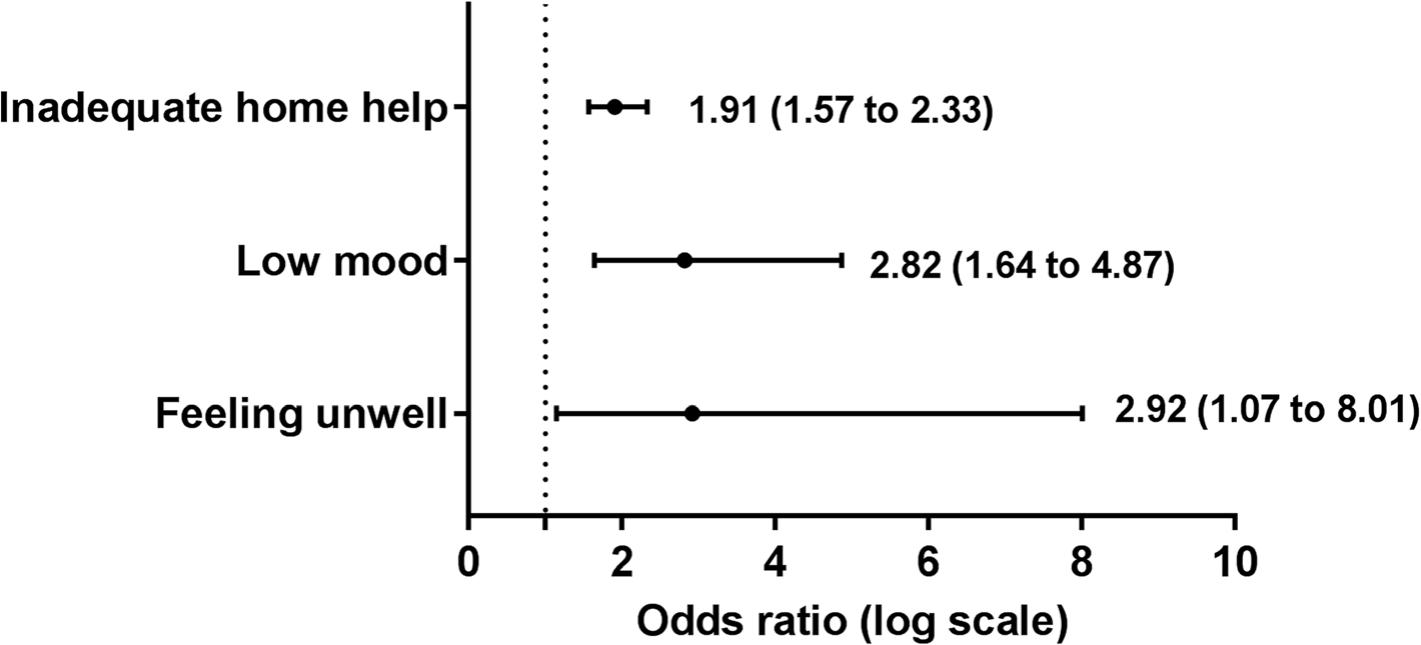
Patient reported barriers associated with low mental health status

Patient reported barriers associated with higher odds of low mental health status included the personal barriers of ‘feeling unwell’ (OR 2.92; 95% CI 1.07 to 8.01), low mood (OR 2.82; 95% CI 1.64 to 4.87) and ‘unavailability of home help’ (OR 1.91; 95% CI 1.57 to 2.33) (Fig. 3 and Additional file 4: Table S3).

Patient reported personal barriers such as socio-economic status and language spoken as well as patient reported clinician and health system barriers such as communication and cost were not associated with lower physical or mental health status (see Additional file 4: Tables S2 and S3).

The greater the total number of patient reported barriers the greater the odds of low mental health status but not physical health status (see Additional file 4: Table S5).

## Discussion

In this multi-site cross sectional study of patients with both diabetes and CKD, patient reported barriers to health care were associated with poorer quality of life. Particularly, the disease having an impact on family and friends, feeling unwell and having other life stressors that make self-care a low priority increased the odds of low physical health status. Additionally, feeling unwell, low mood and difficulty obtaining home help, increased the odds of low mental health status. A greater total number of patient reported barriers was also associated with increased odds of low mental health status.

In our study, the impact of the disease on family and friends was strongly associated with increased odds of low physical health status. This has not been extensively explored in the literature. A qualitative study among patients with comorbid diabetes and CKD has suggested that patients’ tiredness, feeling unwell, increased disability and loss of independence negatively affected their families, marriages and social circles [21]. Consequently, we hypothesise that it is the low physical health status, which has a negative impact on relationships with family and friends, rather than the inverse. This needs to be confirmed in a longitudinal study. Additionally, carer burden and depression has been described especially for those providing care to patients with advanced kidney disease [22–24]. Since there appears to be a direct relationship between family caregivers’ quality of life and that of the patients they care for, it may also be important for the health care system to address the quality of life needs of care givers.

Patients reporting the presence of other life stressors (any other life stressors unrelated to the patients’ illness, family situation and jobs) that made self-care of diabetes and CKD a lower priority was associated with low physical health status. Although not previously studied in patients with both diabetes and CKD, in patients with diabetes alone, lack of engagement in self-care is associated with poorer overall HRQoL [25–28]. Moreover, in patients with CKD alone, self-management programs have been reported to improve mental quality of life measures but not physical quality of life measures [29]. Taken together, these data and our findings suggest that helping patients deal with life stressors so they can better self-care will improve their mental and physical well-being.

Seeing a different doctor in outpatient specialist clinics was associated with lower odds of low physical health status. A possible reason for this is that patients who see a different doctor receive additional opinions or information which may reinforce the information they are provided and improve their perceived health status. In contrast, a study among patients with diabetes showed that consultation by different doctors increased patients’ social vulnerability and directly affected their quality of life [30]. Our findings suggest that different specialists may be used in multidisciplinary clinics such as combined diabetes and kidney clinics without affecting patients’ physical health status.

Additionally, patient reported inadequate diabetes education was associated with lower odds of low physical health status. This was an unexpected finding as patients who have received diabetes education are reported to be more likely to have higher HRQoL [31–33]. An explanation may be that maintaining the impact of diabetes education over time is especially challenging due to competing interests of managing more than one complex disease. Additionally, having inadequate education may mean that patients become less worried or anxious about their health.

Self-reported low mood, which has an impact on motivation to engage in self-management activities [34] was, as expected, associated with lower mental health status. Studies in both CKD and diabetes show an association between low mood and lower scores on quality of life domains of psychological health [35–37]. Here we show that an association similarly exists in patients with both diabetes and CKD. Interventions that screen for and target low mood may result in improved quality of life in this population.

Patients who reported feeling unwell had lower scores for both physical and mental health status in patients with both diabetes and CKD. These associations are intuitive and predictable given the nature of the physical and mental health status scores and serve to validate the rest of our results.

Patient reported difficulty receiving home help was also associated with low mental health status in patients with both diabetes and CKD. As far as we know, this has not been previously reported. This association emphasises the importance of supporting patients with physical disabilities with home help services. Improving access to, and the process of receiving home help, may improve patient quality of life in this group with complex needs.

Finally, we found that a greater number of patient reported barriers was associated with increased odds of low mental health status. This highlights the importance of involving patients in co-designing improvements to health care. This approach makes health services more patient-centred and provides a platform for addressing issues that are important to patients. It also emphasises the importance of addressing these patient reported barriers in health care improvement interventions, as this may lead to improved HRQoL particularly in the mental health domain.

Our findings carry important practice, policy and research implications. First, the approach taken by health services providing care to patients with comorbid diabetes and CKD should consider the barriers to health care for this patient group if physical and mental well-being are to be maintained or even improved. Second, well-being measures may be used to provide information on areas that are less often addressed such as the impact of the disease on family and friends. Additionally, we found that it was possible to assess the patient’s well-being directly in order to tailor interventions appropriately rather than relying on reports from relatives or caregivers. Well designed and disease-specific longitudinal studies are required to determine the impact of patient-reported barriers on patients’ well-being.

Interpretation of our results should be based on the strengths and limitations of the study. Strengths include the multi-site patient recruitment from geographically distinct large metropolitan areas, and the use of a valid and reliable tool to measure HRQoL (SF-12). Limitations include the cross-sectional study design negating our ability to make definitive causal inferences. Thus, the potential for reverse causality cannot be ruled out where low physical and mental well-being may predispose patients to some barriers such as the impact of the disease on family, low mood and feeling unwell. Even though our study excluded non-English speaking patients, we do not think that this would substantially change our findings based on previous studies among patients with diabetes [38, 39]. In addition, we acknowledge that a test–retest reliability was not performed for the patient-reported barriers questionnaire, but partnering with patients in developing this survey ensured a form of reliability in the study. Another limitation is that responders were generally younger and predominantly male with lower eGFR. This finding is in keeping with that of other studies of patients with CKD [40–42].

## Conclusions

Patient reported barriers to health care are associated with both lower physical and mental health status. Additionally, a greater number of patient reported barriers was associated with lower mental health status. Interventions addressing these barriers may improve HRQoL among people with diabetes and CKD.

## Additional files

Additional file 1: DRP: Diabetes Renal Project (Doctors Survey - Health Indicators). (PDF 69 kb)

Additional file 2: Supplementary Appendix S2-Barriers to Health-care Questionnaire. (DOCX 19 kb)

Additional file 3: Kidney Disease and Quality of Life (KDQOL™-36). (PDF 25 kb)

Additional file 4: **Table S1.** Characteristics of patients who did and did not participate in the study. **Table S2.** Univariable and multivariable logistic regression for factors associated with low physical health status (SF Physical Composite Summary <50). **Table S3.** Univariable and multivariable logistic regression for factors associated with low mental health status (SF Mental Composite Summary <50). **Table S4.** Odds of low physical and mental health status by number of patient reported barriers. (DOCX 23 kb)

## Abbreviations

CI: Confidence interval
CKD: Chronic kidney disease
DRP: Diabetes Renal Project
eGFR: Estimated glomerular filtration rate
HRQoL: Health-related quality of life
IRSD: Index of Relative Social Disadvantage
KDQoL: Kidney Disease Quality of life
OR: Odds ratio
PROM: Patient reported outcome measure
SES: Socio economic status
WHO: World Health Organisation
