# Supplementary material for: Patient reported barriers are associated with low physical and mental well-being in patients with co-morbid diabetes and chronic kidney disease
Source: Health Qual Life Outcomes. 2018 Nov 19;16:215. doi: 10.1186/s12955-018-1044-2 (PMC6245917; doi:10.1186/s12955-018-1044-2)
Supplement: Supplementary file 2 — Supplementary Appendix S2-Barriers to Health-care Questionnaire. (DOCX 19 kb) [file 12955_2018_1044_MOESM2_ESM.docx]

**Supplementary Appendix S2**

**Barriers to Health-care Questionnaire**

Sometimes people have difficulty looking after their diabetes and kidney disease due to a variety of barriers or obstacles. Listed below are several barriers that may influence your ability to look after your diabetes and kidney disease. To what extent do you agree or disagree with the following factors being a current barrier for you? (Please tick the appropriate box in the table below)

|  | Disagree | Somewhat Disagree | Somewhat Agree | Agree |
| --- | --- | --- | --- | --- |
| My diabetes and kidney specialist does not spend enough time with me | □ | □ | □ | □ |
| My diabetes and kidney specialist does not provide me with enough information/education about my diabetes and kidney disease | □ | □ | □ | □ |
| I am often seen by a different doctor each time I attend my diabetes or kidney disease appointment. | □ | □ | □ | □ |
| My specialists give me conflicting advice | □ | □ | □ | □ |
| I do not have a good relationship with my specialist or other specialist health service staff. | □ | □ | □ | □ |
| Specialist health service staff are not caring, polite and helpful | □ | □ | □ | □ |
| My specialists do not communicate well with my GP | □ | □ | □ | □ |
| My specialists don’t communicate well with each other | □ | □ | □ | □ |
| I do not have a good GP | □ | □ | □ | □ |
| I need more education and understanding of my diabetes | □ | □ | □ | □ |
| I need more education and understanding of my kidney disease | □ | □ | □ | □ |
| The information provided by my doctors or health professionals is hard to understand because English is not my first language or the information is not culturally relevant | □ | □ | □ | □ |
| The information provided by my doctors or health professionals is too complicated | □ | □ | □ | □ |
| It is difficult to obtain medical support and advice for my diabetes when I need it | □ | □ | □ | □ |
| It is difficult to obtain medical support and advice for my kidney disease when I need it | □ | □ | □ | □ |
| I have had an unsatisfactory prior experience with a diabetes or kidney health service/specialist | □ | □ | □ | □ |
| I am unable to afford the cost of attending appointments or buying medication for my diabetes and kidney disease | □ | □ | □ | □ |
| I have trouble adjusting to the impact that diabetes and kidney disease has made on my life and/or that of my family and friends | □ | □ | □ | □ |
| My diabetes and kidney disease makes me feel very unwell | □ | □ | □ | □ |
| My other illnesses affect my ability to look after my diabetes and kidney disease | □ | □ | □ | □ |
| I have many other stressors in my life, and taking care of my diabetes and kidney disease is not a high priority | □ | □ | □ | □ |
| My job makes it difficult to take care of my diabetes and kidney disease well. | □ | □ | □ | □ |
| My mood (e.g. feeling down, worried, frustrated) gets in the way of me looking after my diabetes and kidney disease | □ | □ | □ | □ |
| I do not feel motivated enough to look after my diabetes and kidney disease well | □ | □ | □ | □ |
| I have trouble maintaining the right diet or fluid restriction for my diabetes and kidney disease | □ | □ | □ | □ |
| I have difficulty knowing what I can eat/drink, for my diabetes and kidney disease | □ | □ | □ | □ |
| I experience unpleasant side-effects from my medication | □ | □ | □ | □ |
| I do not receive support from my family | □ | □ | □ | □ |
| I do not receive support from my friends | □ | □ | □ | □ |
| I find it difficult to get services for home-help | □ | □ | □ | □ |

Do you have difficulty accessing a diabetes service?

□ Yes □ No

Do you have difficulty accessing a kidney service?

□ Yes □ No

.
