# Supplementary material for: Patient reported barriers are associated with low physical and mental well-being in patients with co-morbid diabetes and chronic kidney disease
Source: Health Qual Life Outcomes. 2018 Nov 19;16:215. doi: 10.1186/s12955-018-1044-2 (PMC6245917; doi:10.1186/s12955-018-1044-2)
Supplement: Supplementary file 3 — Kidney Disease and Quality of Life (KDQOL™-36). (PDF 25 kb) [file 12955_2018_1044_MOESM3_ESM.pdf]

Your Health
– and –
Well-Being

Kidney Disease and Quality of Life (KDQOL™-36)

This survey asks for your views about your health. This information will help keep track of how you feel and how well you are able to do your usual activities.

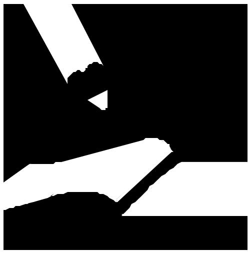

Thank you for completing these questions!

Copyright © 2000 by RAND and the University of Arizona

Study of Quality of Life
For Patients on Dialysis

What is the purpose of the study?

This study is being carried out in cooperation with physicians and their patients. The purpose is to assess the quality of life of patients with kidney disease.

What will I be asked to do?

For this study, we want you to complete a survey today about your health, how you feel and your background.

Confidentiality of information?

We do not ask for your name. Your answers will be combined with those of other participants in reporting the findings of the study. Any information that would permit identification of you will be regarded as strictly confidential. In addition, all information collected will be used only for purposes of the study, and will not be disclosed or released for any other purpose without your prior consent.

How will participation benefit me?

The information you provide will tell us how you feel about your care and further understanding about the effects of medical care on the health of patients. This information will help to evaluate the care delivered.

Do I have to take part?

You do not have to fill out the survey and you can refuse to answer any question. Your decision to participate will not affect your opportunity to receive care.

Your Health

This survey includes a wide variety of questions about your health and your life. We are interested in how you feel about each of these issues.

1. **In general, would you say your health is: [Mark an ☒ in the one box that best describes your answer.]**

Excellent	Very good	Good	Fair	Poor
☐ 1	☐ 2	☐ 3	☐ 4	☐ 5

The following items are about activities you might do during a typical day. <u>Does your health now limit</u> you in these activities? If so, how much? [Mark an ☒ in a box on each line.]

	Yes, limited a lot	Yes, limited a little	No, not limited at all
**2.** <u>Moderate activities</u>, such as moving a table, pushing a vacuum cleaner, bowling, or playing golf	☐ 1	☐ 2	☐ 3
**3.** Climbing <u>several</u> flights of stairs	☐ 1	☐ 2	☐ 3

During the <u>past 4 weeks</u>, have you had any of the following problems with your work or other regular daily activities <u>as a result of your physical health</u>?

	Yes	No
**4.** <u>Accomplished less</u> than you would like...............	☐ 1	☐ 2
**5.** Were limited in the <u>kind</u> of work or other activities..	☐ 1	☐ 2

During the past 4 weeks, have you had any of the following problems with your work or other regular daily activities as a result of any emotional problems (such as feeling depressed or anxious)?

	Yes	No
**6.** <u>Accomplished less</u> than you would like...............	☐ 1	☐ 2
**7.** Didn't do work or other activities as <u>carefully</u> as usual...	☐ 1	☐ 2

8. During the <u>past 4 weeks</u>, how much did <u>pain</u> interfere with your normal work (including both work outside the home and housework)?

Not at all	A little bit	Moderately	Quite a bit	Extremely
☐ 1	☐ 2	☐ 3	☐ 4	☐ 5

These questions are about how you feel and how things have been with you <u>during the past 4 weeks</u>. For each question, please give the one answer that comes closest to the way you have been feeling.

How much of the time during the <u>past 4 weeks</u>…

	All of the time	Most of the time	A good bit of the time	Some of the time	A little of the time	None of the time
**9.** Have you felt calm and peaceful?	☐ 1	☐ 2	☐ 3	☐ 4	☐ 5	☐ 6
**10.** Did you have a lot of energy?	☐ 1	☐ 2	☐ 3	☐ 4	☐ 5	☐ 6
**11.** Have you felt downhearted and blue?	☐ 1	☐ 2	☐ 3	☐ 4	☐ 5	☐ 6

12. During the <u>past 4 weeks</u>, how much of the time has your <u>physical health or emotional problems</u> interfered with your social activities (like visiting with friends, relatives, etc.)?

All of the time	Most of the time	Some of the time	A little of the time	None of the time
☐ 1	☐ 2	☐ 3	☐ 4	☐ 5

Your Kidney Disease

How true or false is each of the following statements for you?

	Definitely true	Mostly true	Don't know	Mostly false	Definitely false
**13.** My kidney disease interferes too much with my life	☐ 1	☐ 2	☐ 3	☐ 4	☐ 5
**14.** Too much of my time is spent dealing with my kidney disease	☐ 1	☐ 2	☐ 3	☐ 4	☐ 5
**15.** I feel frustrated dealing with my kidney disease	☐ 1	☐ 2	☐ 3	☐ 4	☐ 5
**16.** I feel like a burden on my family	☐ 1	☐ 2	☐ 3	☐ 4	☐ 5

During the <u>past 4 weeks</u>, to what extent were you bothered by each of the following?

		Not at all bothered	Somewhat bothered	Moderately bothered	Very much bothered	Extremely bothered
**17.**	Soreness in your muscles?	☐ 1	☐ 2	☐ 3	☐ 4	☐ 5
**18.**	Chest pain?	☐ 1	☐ 2	☐ 3	☐ 4	☐ 5
**19.**	Cramps?	☐ 1	☐ 2	☐ 3	☐ 4	☐ 5
**20.**	Itchy skin?	☐ 1	☐ 2	☐ 3	☐ 4	☐ 5
**21.**	Dry skin?	☐ 1	☐ 2	☐ 3	☐ 4	☐ 5
**22.**	Shortness of breath?	☐ 1	☐ 2	☐ 3	☐ 4	☐ 5
**23.**	Faintness or dizziness?	☐ 1	☐ 2	☐ 3	☐ 4	☐ 5
**24.**	Lack of appetite?	☐ 1	☐ 2	☐ 3	☐ 4	☐ 5
**25.**	Washed out or drained?	☐ 1	☐ 2	☐ 3	☐ 4	☐ 5
**26.**	Numbness in hands or feet?	☐ 1	☐ 2	☐ 3	☐ 4	☐ 5
**27.**	Nausea or upset stomach?	☐ 1	☐ 2	☐ 3	☐ 4	☐ 5
**28[a].**	(Hemodialysis patient only) Problems with your access site?	☐ 1	☐ 2	☐ 3	☐ 4	☐ 5
**28[b].**	(Peritoneal dialysis patient only) Problems with your catheter site?	☐ 1	☐ 2	☐ 3	☐ 4	☐ 5

Effects of Kidney Disease on Your Daily Life

Some people are bothered by the effects of kidney disease on their daily life, while others are not. How much does kidney disease <u>bother</u> you in each of the following areas?

	Not at all bothered	Somewhat bothered	Moderately bothered	Very much bothered	Extremely bothered
**29.** Fluid restriction?	☐ 1	☐ 2	☐ 3	☐ 4	☐ 5
**30.** Dietary restriction?	☐ 1	☐ 2	☐ 3	☐ 4	☐ 5
**31.** Your ability to work around the house?	☐ 1	☐ 2	☐ 3	☐ 4	☐ 5
**32.** Your ability to travel?	☐ 1	☐ 2	☐ 3	☐ 4	☐ 5
**33.** Being dependent on doctors and other medical staff?	☐ 1	☐ 2	☐ 3	☐ 4	☐ 5
**34.** Stress or worries caused by kidney disease?	☐ 1	☐ 2	☐ 3	☐ 4	☐ 5
**35.** Your sex life?	☐ 1	☐ 2	☐ 3	☐ 4	☐ 5
**36.** Your personal appearance?	☐ 1	☐ 2	☐ 3	☐ 4	☐ 5

Thank you for completing these questions!
