# Supplementary material for: Patient reported barriers are associated with low physical and mental well-being in patients with co-morbid diabetes and chronic kidney disease
Source: Health Qual Life Outcomes. 2018 Nov 19;16:215. doi: 10.1186/s12955-018-1044-2 (PMC6245917; doi:10.1186/s12955-018-1044-2)
Supplement: Supplementary file 4 — Table S1. Characteristics of patients who did and did not participate in the study. Table S2. Univariable and multivariable logistic regression for factors associated with low physical health status (SF Physical Composite Summary <50). Table S3. Univariable and multivariable logistic regression for factors associated with low mental health status (SF Mental Composite Summary <50). Table S4. Odds of low physical and mental health status by number of patient reported barriers. (DOCX 23 kb) [file 12955_2018_1044_MOESM4_ESM.docx]

**Additional file 4**

**S1**: Characteristics of patients who did and did not participate in the study

|  | **Responders** | **Non-responders** | **p-value** |
| --- | --- | --- | --- |
| Patient numbers (n) | 308 | 120* | - |
| Age (SD) | 66.9 (11.0) | 71.5 (9.6) | 0.0001 |
| Gender (Female, %) | 30.5 | 40.8 | 0.04 |
| Type of diabetes (Type 2, %) | 88.0 | 94.2 | 0.06 |
| eGFR, mean (SD) | 29.0 (16.7) | 36.3 (10.0) | 0.0001 |

* Non-responders from one sampling site; eGFR was calculated using the Chronic Kidney Disease Epidemiology Collaboration equation and expressed in mL/min/1.73 m^2^

**S2**: Univariable and multivariable logistic regression for factors associated with low physical health status (SF Physical Composite Summary <50)

|  | **Univariable** | | **Multivariable** | |
| --- | --- | --- | --- | --- |
| Factor | OR (95% CI) | p-value | OR (95% CI) | p-value |
| Age | 1.00 (0.98 to 1.03) | 0.72 | 1.04 (1.02 to 1.06) | 0.001 |
| Gender (Ref: male) | 2.60 (1.74 to 3.89) | 0.001 | 1.44 (0.69 to 3.00) | 0.33 |
| Socioeconomic status | 1.00 (0.99 to 1.01) | 0.40 | - | - |
| Language (Ref: English) | 4.9 (1.25 to 19.39) | 0.02 | 3.05 (0.75 to 12.40) | 0.12 |
| Inadequate time spent with specialist | 0.92 (0.49 to 1.72) | 0.79 | - | **-** |
| Inadequate information provided by specialist | 1.20 (0.43 to 3.34) | 0.72 | - | **-** |
| Being seen by a different doctor | 0.71 (0.48 to 1.04) | 0.08 | 0.47 (0.27 to 0.80) | 0.01 |
| Poor relationship with health staff | 3.80 (0.24 to 60.13) | 0.34 | - | - |
| Staff not caring, polite and helpful | 2.23 (0.19 to 26.64) | 0.53 | - | - |
| Poor communication from specialists to GPs | 1.62 (0.41 to 6.35) | 0.49 | - | - |
| Poor communication between specialists | 1.34 (1.06 to 1.69) | 0.01 | 1.44 (0.71 to 2.92) | 0.32 |
| Not having a good GP | 1.37 (0.46 to 4.05) | 0.57 | - | - |
| Inadequate diabetes education | 0.68 (0.58 to 0.79) | 0.001 | 0.40 (0.22 to 0.72) | 0.002 |
| Inadequate kidney disease education | 0.95 (0.23 to 3.99) | 0.95 | - | - |
| Complicated education material | 1.15 (0.61 to 2.14) | 0.67 | - | - |
| Inadequate advice regarding diabetes | 1.82 (0.63 to 5.25) | 0.27 | - | - |
| Inadequate advice regarding kidney disease | 1.09 (0.29 to 4.07) | 0.90 | - | - |
| Unsatisfactory previous experience | 0.64 (0.36 to 1.14) | 0.13 | - | - |
| Costs (transport and buying medications) | 1.42 (0.46 to 4.36) | 0.54 | - | - |
| Impact of disease on family and friends | 3.48 (1.71 to 7.07) | 0.001 | 2.07 (1.14 to 3.78) | 0.02 |
| Feeling unwell due to illness | 3.83 (1.25 to 11.8) | 0.02 | 4.23 (1.45 to 12.30) | 0.01 |
| Other illness | 5.51 (0.62 to 49.0) | 0.13 | - | - |
| Having other life stressors | 3.59 (1.97 to 6.54) | 0.001 | 2.59 (1.20 to 5.61) | 0.02 |
| Mood affects self-management | 2.71 (1.06 to 6.95) | 0.04 | 0.98 (0.57 to 1.66) | 0.93 |
| Feels unmotivated to self-manage | 1.51 (0.36 to 6.35) | 0.57 | - | - |
| Maintaining dietary and fluid restrictions | 1.67 (0.81 to 3.46) | 0.17 | - | - |
| Not knowing what is allowed to eat/drink | 1.47 (0.46 to 4.74) | 0.52 | - | - |
| Experience of medication side effects | 3.60 (2.08 to 6.25) | 0.001 | 1.73 (0.53 to 5.62) | 0.36 |
| Inadequate support from family | 2.63 (0.97 to 7.13) | 0.06 | - | - |
| Inadequate support from friends | 3.89 (1.12 to 13.58) | 0.03 | 2.84 (0.5 to 15.67) | 0.23 |
| Difficulties getting home help | 2.87 (1.27 to 6.50) | 0.01 | 1.40 (0.92 to 2.12) | 0.12 |

Variable with a P<0.05 were included in the logistic multivariable model. Variables with P<0.05 in logistic multivariable were significant. GP-General Practitioner; OR-Odds ratio; CI-Confidence interval.

**S3**: Univariable and multivariable logistic regression for factors associated with low mental health status (SF Mental Composite Summary <50)

|  | **Univariable** | | **Multivariable** | |
| --- | --- | --- | --- | --- |
| Factor | OR (95% CI) | p-value | OR (95% CI) | p-value |
| Age | 0.97 (0.96 to 0.98) | 0.001 | 0.99 (0.98 to 1.00) | 0.20 |
| Gender (Ref: male) | 1.11 (1.01 to 1.22) | 0.03 | 1.15 (0.65 to 2.05) | 0.62 |
| Socioeconomic status | 1.00 (0.99 to 1.01) | 0.04 | 1.00 (0.99 to 1.01) | 0.26 |
| Language (Ref: English) | 1.72 (0.95 to 3.11) | 0.07 | - | - |
| Inadequate time spend with specialist | 2.17 (1.17 to 4.04) | 0.01 | 1.67 (0.53 to 5.27) | 0.39 |
| Specialist provides inadequate information | 2.23 (0.96 to 5.14) | 0.06 | - | - |
| Being seen by different specialists | 1.50 (1.40 to 1.66) | 0.001 | 1.02 (0.48 to 2.17) | 0.95 |
| Poor relationship with health staff | 3.00 (1.46 to 6.18) | 0.001 | 2.37 (0.80 to 7.04) | 0.12 |
| Staff not caring, polite and helpful | 1.94 (1.46 to 2.56) | 0.001 | 0.52 (0.25 to 1.10) | 0.09 |
| Poor communication from specialists to GPs | 1.44 (0.85 to 2.46) | 0.18 | - | - |
| Poor communication between specialists | 2.02 (1.16 to 3.52) | 0.01 | 1.12 (0.46 to 2.73) | 0.80 |
| Not having a good GP | 3.85 (1.19 to 12.51) | 0.03 | 2.18 (0.68 to 7.03) | 0.19 |
| Inadequate diabetes education | 0.81 (0.45 to 1.48) | 0.50 | - | - |
| Inadequate kidney disease education | 0.98 (0.64 to 1.52) | 0.94 | - | - |
| Complicated education material | 1.24 (0.83 to 1.84) | 0.29 | - | - |
| Inadequate advice regarding diabetes | 1.23 (0.31 to 4.89) | 0.77 | - | - |
| Inadequate advice regarding kidney disease | 1.74 (0.52 to 5.84) | 0.37 | - | - |
| Unsatisfactory previous experience | 1.53 (0.62 to 3.80) | 0.36 | - | - |
| Costs (transport and buying medications) | 1.48 (1.04 to 2.10) | 0.03 | 0.59 (0.32 to 1.07) | 0.08 |
| Disease affecting family and friends | 3.36 (1.96 to 5.76) | 0.001 | 1.16 (0.90 to 1.49) | 0.25 |
| Feeling unwell due to illness | 3.82 (1.85 to 7.92) | 0.001 | 2.92 (1.07 to 8.01) | 0.04 |
| Other illness | 4.39 (1.47 to 13.16) | 0.01 | 1.47 (0.29 to 7.32) | 0.64 |
| Having other life stressors | 2.50 (1.67 to 3.73) | 0.001 | 1.16 (0.45 to 3.00) | 0.76 |
| Mood affects self-management | 7.29 (3.66 to 14.51) | 0.001 | 2.82 (1.64 to 4.87) | 0.001 |
| Feels unmotivated to self-manage | 4.19 (1.47 to 11.94) | 0.01 | 1.54 (0.43 to 5.57) | 0.51 |
| Maintaining dietary and fluid restrictions | 1.97 (1.20 to 3.24) | 0.01 | 0.78 (0.41 to 1.50) | 0.46 |
| Not knowing what is allowed to eat/drink | 1.27 (0.73 to 2.23) | 0.40 | - | - |
| Experience of medication side effects | 2.53 (1.85 to 3.46) | 0.001 | 0.96 (0.48 to 1.93) | 0.91 |
| Inadequate support from friends | 3.18 (1.89 to 5.34) | 0.001 | 1.66 (0.24 to 11.67) | 0.61 |
| Inadequate support from family | 3.36 (1.22 to 9.26) | 0.02 | 1.95 (0.84 to 4.55) | 0.12 |
| Difficulties getting home help | 3.44 (2.11 to 5.61) | 0.001 | 1.91 (1.57 to 2.33) | 0.001 |

Variable with a P<0.05 were included in the logistic multivariable model. Variables with P<0.05 in logistic multivariable were significant. GP-General Practitioner; OR-Odds ratio; CI-Confidence interval.

**Table S4:** Odds of low physical and mental health status by number of patient reported barriers

| **Number of barriers*** | **Low physical health scores** |  | **Low mental health scores** |  |
| --- | --- | --- | --- | --- |
|  | OR (95% CI) | P-value | OR (95% CI | P-value |
| 0-3 (reference) |  |  |  |  |
| 4-8 | 1.67 (0.64 to 4.36) | 0.29 | 2.03 (1.52 to 2.69) | 0.001 |
| 9-29 | 1.87 (0.61 to 5.75) | 0.28 | 4.08 (1.43 to 11.61) | 0.01 |

* The total number of barriers identified by each patient were categorised into tertiles. The first tertile (patient reported 0-3 barriers) was the reference group; OR-Odd ratio; CI-Confidence interval
